# From Copenhagen chill to desert heat thrill

**DOI:** 10.1113/EP092410

**Published:** 2025-01-24

**Authors:** Lars Nybo

**Affiliations:** ^1^ Department of Nutrition, Exercise and Sport Sciences University of Copenhagen Copenhagen Denmark

1

I am in the Qatari desert, half an hour into an experimental 43 km cycling time trial (TT), already overcooked and thinking: ‘Why did the idiotic researcher design this experiment? Lars, what were you thinking exposing unacclimatized cyclists, including yourself, to exhaustive exercise in a 35°C hot desert?’. Only 15 min earlier, I was feeling good; it was our first day in the sun, my legs were good, my speed high and my feeling similar to the cool control TT we completed in Copenhagen before heading to Qatar for the field studies with the PhD and two master students I was supervising. Together with Sebastian Racinais, whom I had completed heat studies with a few years before this project (e.g., Mohr et al., 2012; Nybo et al., 2013) and lead author of several consensus papers on training in the heat (e.g., Racinais et al., 2015), we had planned and organized the study, with the aim being to study ‘Time course of natural heat acclimatization in well‐trained cyclists during a 2‐week training camp in the heat’ (Karlsen, Nybo et al., 2015) and also to evaluate whether the heat acclimatization, as indicated by work from Lorenzo et al. (2010), would translate into improved performance in cooler settings (Karlsen, Racinais et al., 2015). The day before the TT, we had completed a standard laboratory protocol at 44°C (0.5 h exercise at 140 W followed by 0.5 h seated rest to study sweat loss and thermal responses to standard submaximal exposure), and I felt good throughout the 1 h laboratory exposure, like I normally do when we complete laboratory studies in the heat.

I have, since my first study on performance effects of hyperthermia and dehydration (Nybo et al., 2001; which was also my master project supervised by Bodil Nielsen and Gonzalez‐Alonso) and in the majority of my previous heat studies, most often in the pilot phase, ‘enjoyed’ being the subject, to experience the physiology on my own body before exposing other participants to the heat and other demanding study protocols. In the present project, as a participant in the real study, I fulfilled the inclusion criteria: at that time competing in national elite cycling, annually training >20,000 km and completing regular high‐intensity training. This pre‐season, however, all sessions were completed in cool settings (outdoors, where the air temperature had been <5°C all winter ahead of the study) to comply with the criteria of not being acclimatized or acclimated ahead of the trip to Qatar. So here I was, not even halfway through the TT and, as revealed by the analyses completed afterward (and shown by red circles in Figure 1), already with a core temperature of ∼40°C. We/I were obviously aware that the heat‐TT in the unacclimatized state would be a hard and fatiguing challenge, but we (in fact, me personally) had instructed the participants to do their best and complete the trial no matter how fatigued or bad they would feel. So, I kept struggling, although my power, speed and energy were gone. I was what we in cycling would call a cold camel (paradoxical expression considering I was overcooked). Like a wrecked ship in the desert, I was in survival mode and eventually completed the last 30 min with a power output ∼100 W lower than the first 15 min when I was feeling good and apparently did not obtain afferent feedback relevant for early adjustment of the intensity. The initial power was a pace I was capable of maintaining for 1 h in cool settings, but apparently a too optimistic pacing strategy that all participants experienced in this unacclimatized TT [compare red circles (my individual response) with the mean of other participants, as shown with a continuous black line in Figure 1; see also Racinais, Périard et al., 2015)]. However, in contrast to the other participants, in whom the reduced work rate, hence lower endogenous metabolic heat production, allowed them to complete the last part of the TT without further heat storage and core temperature stabilizing at ∼40°C, my core temperature went crazy. Forget about ‘anticipatory regulation and avoidance of catastrophe during exercise‐induced hyperthermia’ (Marino, 2004). Although ending with an internal temperature of 41.1°C, I was feeling cold at the first signs of exertional heat stroke and spent the following 2 days with nausea and a bad stomach as signs of intestinal barrier dysfunction and endotoxaemia (Laitano et al., 2019).

**FIGURE 1:**
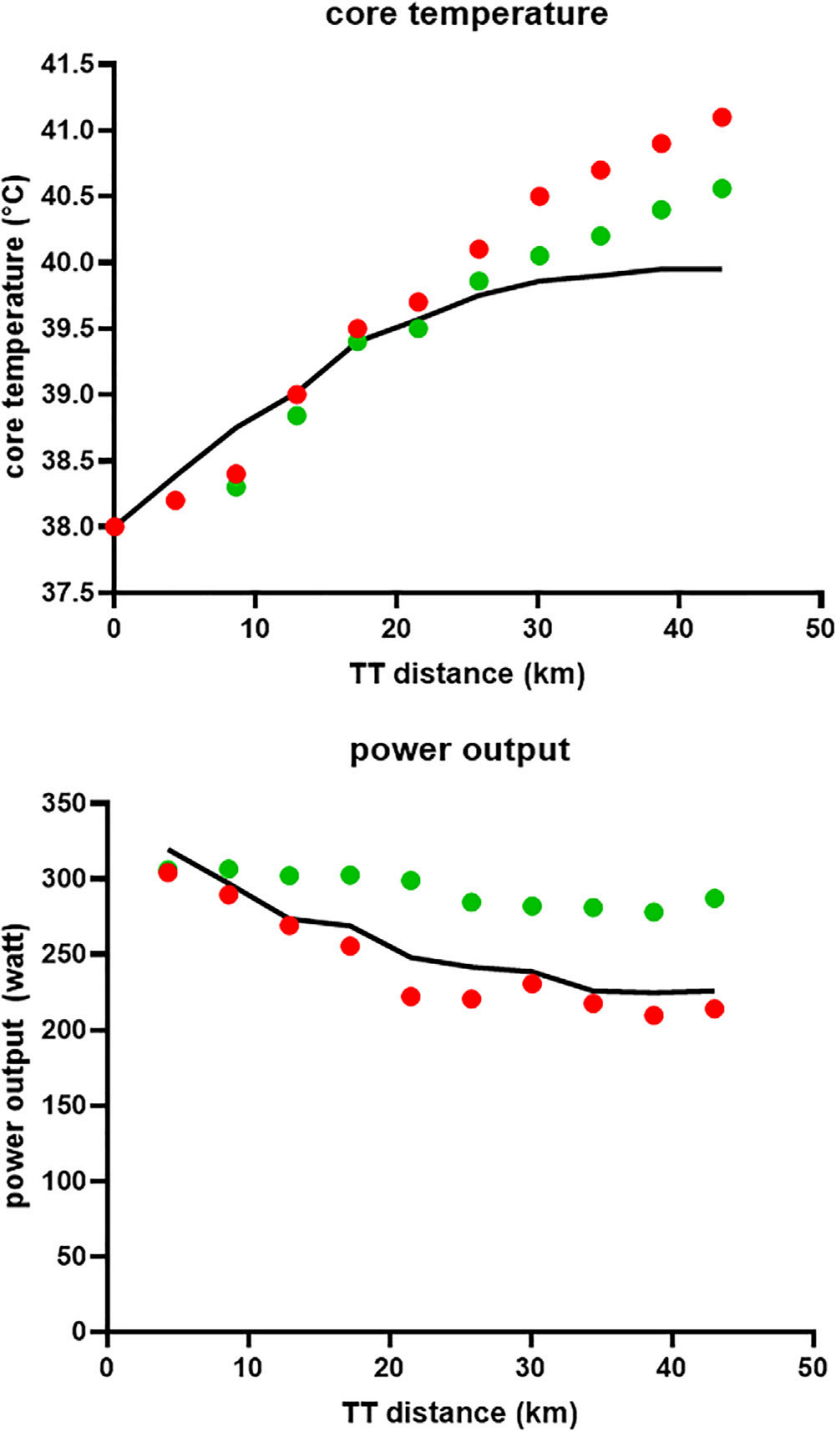
The top panel shows core temperature responses (measured with an intestinal pill; *y*‐axis, in degrees Celsius) during the first TT (*x*‐axis, in kilometres) completed in the unacclimatized state (red circles representing the case subject and the black line the average response for the remaining participants) and the response from the third TT (green circles for the case subject) completed after 2 weeks of acclimatization to the ∼35°C (wet bulb globe temperature of 28°C) environment, that is, the heat conditions for the TTs in addition to the daily training sessions between the two experimental time trials. The bottom panel shows the power output (*y*‐axis, in watts) during the first TT (red circles representing the case subject and black lines the average response for the remaining participants), and the green circles are the power output for the TT completed after acclimatization. Each circle represents the average value for 10% of the TT segment. Abbreviation: TT, time trial.

During the following days, I struggled to complete easy heat training sessions and also kept feeling ill off the bike, so I worried that the entire 14 days would be impossible to complete and certainly with no prospect of doing well in the next TT (which was approaching fast and rescheduled to 1 day earlier owing to a forecast sandstorm; leaving me with a total of only 5 days of heat acclimatization from my first fatal TT). However, heat adaptation is, on some parameters, a miraculously fast process, and already on day 4, I started feeling better. In the second TT (completed on day 6 in the desert), I finished the 43 km at the same speed as in cool Copenhagen and with 50 W higher average power compared with the first heat TT. With 3 W further gain from the second to the third TT (after 14 days acclimatization; green dots in Figure 1), I was 3 min faster than the pre‐heat camp (control) TT. My power was still ∼10 W lower on average compared with cool settings, but in cycling, you go faster in the heat because the air density/resistance is lower (Nybo, 2010). In the last TT I had a slightly lower end core temperature (see Figure 1), but in the second TT (where we only had pre/post measures) I also had an end‐exercise core temperature >41°C, as reported in several cases following TT cycling in high heat scenarios (Racinais et al., 2019). But in both instances, I had no pathophysiological responses, only feeling tired from going as hard as possible. Heat acclimatization certainly works and is essential if you want to perform well and not become ill from competing in hot venues.

Incentivized by the marked improvements in performance during the heat camp, I returned to Copenhagen expecting that all the hard work, sweat and lost salt (plus ditto ingested; I am a salty sweater, as indicated by the picture of my shoes in Figure 2 and in this study with ∼80 mM [Na^+^] measured in my sweat in the acclimatized state) would enhance my TT performance in a cool setting, translating to a gain in maximal O_2_ uptake, as the promising results by Lorenzo et al. (2010) had indicated. However, although we tested both TT performance and maximal O_2_ uptake on two occasions on separate days, ∼1 and ∼2 weeks after return, to ensure that post‐camp fatigue or travel did not preclude us from detecting effects, we saw no transfer effect. Similar findings were reported later by Lundby and co‐workers (Keiser et al., 2015) following indoor acclimatization. The studies and counterpoint discussions with Minson and Cotter (2016) did, however, prompt us to initiate prolonged heat training studies (with 5 weeks of heat acclimation, in comparised to the 2 week exposure here; see Mikkelsen et al., 2019; Oberholzer et al., 2019). Those studies, in addition to recent studies with assessment of blood volume after 3 and 5 weeks (e.g., Cubel et al., 2024), have taught us that if the heat acclimation period is prolonged even trained athletes can boost their haemoglobin mass and improve performance in a cool setting by training in the heat. Therefore, although the 14 days in the Qatari dessert felt like a really long time (and I was ready to go home after only 0.5 h in the heat), we should probably have stayed for at least a couple of weeks more to gain the full effect. But we learned our lessons from the study, and although my personal part was unpleasant and certainly not planned, it adds to my lived physiological experiences and my respect for the major impact that heat can have on our physiology.

**FIGURE 2:**
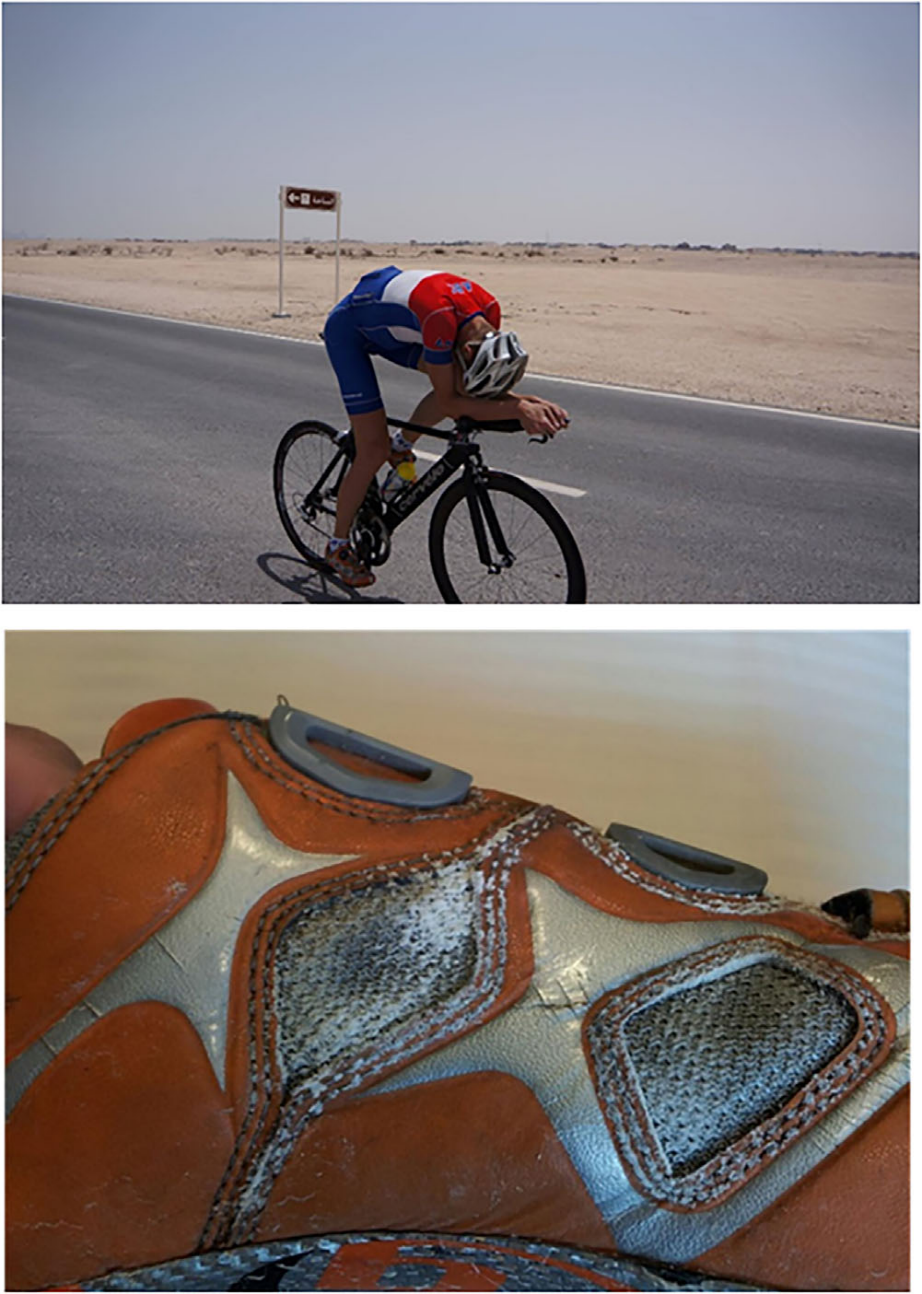
Top picture from the time trial, with an attempt to minimize the frontal area to pass as easily as possible through the hot air, but even with less dense air and faster speed for a given power, the effects of heat were devastating for the unacclimatized cyclist. Lower picture shows glistening salt accumulated in the shoes of the case participant, signifying the massive salt losses experienced during the 2 weeks of training in the heat, where the estimated sweat loss of ∼5 L/day with [Na^+^] of ∼80 mM implied daily losses of >20 g of salt.

## AUTHOR CONTRIBUTIONS

Sole author.

## CONFLICT OF INTEREST

The author declares no conflict of interest.

## FUNDING INFORMATION

None.
